# Anthelmintic Activity of Traditional Medicinal Plants Used in Europe

**DOI:** 10.3390/biology14121636

**Published:** 2025-11-21

**Authors:** Olexandra Boyko, Viktor Brygadyrenko

**Affiliations:** 1 Department of Parasitology and Veterinary Expertise, Examination, Faculty of Veterinary Medicine, Dnipro State Agrarian and Economic University, Sergiy Efremov St., 25, 49000 Dnipro, Ukraine; 2 Department of Biodiversity and Ecology, Faculty of Biology and Ecology, Oles Honchar Dnipro National University, Nauky Av., 72, 49010 Dnipro, Ukraine

**Keywords:** *Strongyloides papillosus*, *Haemonchus contortus*, medicinal plants, nematicidal activity

## Abstract

Synthetic anthelmintic drugs not only contribute to the development of pathogen resistance and environmental pollution but also to the development of pathogen resistance. Therefore, identifying the anthelmintic properties of widely used medicinal plants could be of great practical interest to veterinary and human medicine. In our experiment, we evaluated the in vitro survivability of the noninvasive and invasive (L_1–2_ and L_3,_ respectively) larvae of *Strongyloides papillosus* and *Haemonchus contortus* subject to aqueous solutions of ethanolic tinctures of traditional medicinal plants (46 species). Most of the plant species we studied belonged to the families Asteraceae (*Achillea millefolium*, *Arctium lappa*, *Artemisia absinthium*, *Bidens tripartita*, *Calendula officinalis*, *Cynara cardunculus* var. *scolymus*, *Echinacea purpurea*, *Helichrysum arenarium*, *Inula helenium*, *Matricaria chamomilla*, *Silybum marianum*, *Tanacetum vulgare*, *Taraxacum officinale*, *Tragopogon porrifolius*), Rosaceae (*Agrimonia eupatoria*, *Fragaria vesca*, *Sanguisorba officinalis*), and Lamiaceae (*Leonurus cardiaca*, *Mentha × piperita*, *Origanum vulgare*, *Salvia officinalis*, *Thymus vulgaris*). Other plant families were represented by 1–3 species: Fabaceae (*Glycyrrhiza glabra*, *Hedysarum alpinum*, *Trifolium pratense*), Salicaceae (*Populus nigra*, *P. tremula*, *Salix alba*), Fagaceae (*Quercus robur*), Betulaceae (*Betula pendula*), Juglandaceae (*Juglans regia*), Rhamnaceae (*Frangula alnus*), Acoraceae (*Acorus calamus*), Apiaceae (*Foeniculum vulgare*), Caprifoliaceae (*Valeriana officinalis*), Cucurbitaceae (*Cucurbita pepo*), Equisetaceae (*Equisetum arvense*), Ericaceae (*Vaccinium vitis-idaea*), Gentianaceae (*Centaurium erythraea*), Hypericaceae (*Hypericum perforatum*), Malvaceae (*Althaea officinalis*), Plantaginaceae (*Linaria vulgaris*, *Plantago major*), Poaceae (*Zea mays*), Polygonaceae (*Polygonum aviculare*), and Ranunculaceae (*Nigella sativa*). We determined *Artemisia absinthium*, *Inula helenium*, *Matricaria chamomilla*, *Salvia officinalis*, and *Populus nigra*, whose aqueous solutions of alcohol tinctures demonstrated nematocidal properties. The other plants we studied did not affect the viability of parasitic nematode larvae.

## Simple Summary

Each year, large and small animal-farming complexes and farms suffer significant economic losses due to parasitic diseases of agricultural animals. Losses include reduced meat and dairy productivity, as well as expenses for animal treatment. The intensification of the use of synthetic anthelmintic drugs promotes not only the development of resistance in the pathogens but also a large-scale contamination of the environment with those drugs. All this underlines the necessity of developing new, alternative, natural-origin means of combating parasites that do not accumulate in the environment and contaminate food chains. In our experiment, we evaluated the in vitro survivability of the noninvasive and invasive larvae of *Strongyloides papillosus* and *Haemonchus contortus* subject to aqueous solutions of ethanolic tinctures of traditional medicinal plants (46 species). We determined the representatives of the families Asteraceae (*Artemisia absinthium*, *Inula helenium*, *Matricaria chamomilla*), Lamiaceae (*Salvia officinalis*), and Salicaceae (*Populus nigra*), whose aqueous solutions of alcohol tinctures demonstrated nematocidal properties.

## 1. Introduction

For many centuries, Europeans have been using various plants in the traditional treatment of a broad spectrum of ailments. Parasitic diseases, including helminthiasis, cause significant economic losses to livestock farms. Some of the most common nematodes of the ruminant digestive tract are *Strongyloides papillosus* (Wedl, 1856) Ransom, 1911 (Strongyloididae) and *Haemonchus contortus* (Rudolphi, 1803) Cobb, 1898 (Trichostrongylidae) [1,2]. As of now, the resistance of pathogens of parasitic diseases to synthetic drugs is growing, including pathogens of gastrointestinal nematodes [3,4], and therefore natural compounds garner great interest as alternatives [5,6,7].

Thus, over recent years, the search for effective means against parasitic diseases has been concentrated on evaluating properties of medicinal plants and their compounds as alternative antiparasitic drugs [8,9,10]. Thus, *Artemisia absinthium* L. has antifungal, fungicidal, antimicrobial, insecticidal, acaricidal, and antiprotozoal properties against *Leishmania aethiopica* and *L. donovani* [11,12,13]. *Bidens tripartita* L. also has antimalarial and other antiparasitic properties [14,15]. *Matricaria chamomilla* L. plants have various medicinal properties, including antiparasitic ones [16,17]. Baranauskienė et al. [18] write about the anthelmintic properties of *Tanacetum vulgare* L. Other species of this genus also have many useful medicinal properties, including antiparasitic properties [19,20].

The plant species *Silybum marianum* (L.) has many medicinal properties, as well as antiparasitic activity [21,22]. The medicinal properties, including antiparasitic, of the bark of *Quercus robur* L. are reported in the works of Moharram et al. [23], Fakour & Meshgi [24], and Şöhretoğlu & Renda [25]. Many other scientific works have been published to date, devoted to the medicinal properties of plants, including their antiparasitic activity [26,27,28].

Therefore, one alternative to anthelmintics, including those treating nematodiases of ruminants, is the use of traditional medicinal plants that contain bioactive compounds. The objective of our study was to explore 46 species of medicinal plants that are common in the steppe and forest-steppe zones of Ukraine, as well as Central Europe, by testing their ethanolic extracts for in vitro effects on the survival of the nematodes *S. papillosus* and *H. contortus*.

## 2. Materials and Methods

For the experiment, we purchased the aboveground and underground parts of medicinal plants (Liktravy Ltd., Zhytomyr, Ukraine): roots, rhizomes, bark, aboveground parts, buds, leaves, inflorescences, pericarp, stamens, fruits, and seeds. A total of 46 traditional medicinal plants were used in the experiment. In 30 days, we prepared 10% alcoholic tinctures (2.7 g of ethanol + 0.3 g of dry, fragmented parts of plants) stored at room temperature in the absence of light, periodically shaking them. To do this, the required part of the plant was chopped to 2 mm with scissors and filled with ethanol.

The survival of the nematodes subject to medicinal plants was determined using 0.01%, 0.1%, and 1.0% concentrations of ethanolic tinctures: to 0.01, 0.1, or 1.0 mL of 10% ethanolic tincture, we added 9.99, 9.9, or 9.0 mL of H_2_O. Negative (distilled water) and positive controls (Albendazole 10%, Ukrvetbiopharm, Kharkiv, Ukraine) were also used.

Feces were collected from goats naturally infected by the nematodes *S. papillosus* and *H. contortus*. The study used various larval stages of nematodes that develop in the environment, outside the host organism. This choice was based on the different sensitivities of larvae at different ages to environmental factors. The nematode larvae were cultivated for 10 days at a temperature of 18–22 °C. Cultivation was carried out according to the method of Zajac & Conboy [29]. For this purpose, fresh (1 day) goat feces in their original form, without grinding, were placed in regularly aerated Petri dishes and moistened daily with 3 mL of water at room temperature. The larvae were detected with the generally accepted Baermann’s Method [29] using a centrifuge (1500 rpm for four minutes, 252 g). The larvae of the nematodes *S. papillosus* and *H. contortus* of different ages were identified according to the morphological traits [30,31,32,33].

After centrifugation, 0.1 mL of the sediment with nematode larvae was placed in 1.5 mL plastic test tubes. To 0.1 mL of the sediment with nematode larvae, we added 1 mL of an aqueous solution of ethanolic tincture of plants in five repetitions at 22 °C. The exposure lasted for 24 h. Afterward, the sediment (0.1 mL from each test tube) was placed on a microscope slide to count live and dead larvae (immobile, with signs of damaged intestines).

According to the results of the experiment, we calculated the mean and standard deviation (x ± SD). Statistically significant differences were considered to be *p* ˂ 0.05. Significance of the differences between the samples was evaluated using the ANOVA. To determine the differences between the samples within one line in tables, we used Tukey’s Test.

## 3. Results

The studies of the effects of the medicinal plants on the survival of the nematode larvae revealed the representatives of the families Asteraceae, Lamiaceae, and Salicaceae, whose alcoholic tinctures demonstrated nematocidal properties. Such plants representing the Asteraceae family included *Artemisia absinthium* L., *Inula helenium* L., and *Matricaria chamomilla* L. (Table 1). Over 65% of the noninvasive larvae of *S. papillosus* died within a day when exposed to the alcoholic tinctures of these plants. Meanwhile, the invasive larvae of these nematodes (L_3_ *S. papillosus* and *H. contortus*) were more resistant to these tinctures. Only *I. helenium* displayed a nematocidal activity toward *H. contortus*: around 57% dead larvae were recorded.

Pronounced nematocidal properties against the free-living (nonparasitic) forms of *S. papillosus* were also exerted by a representative of the Lamiaceae family—*Salvia officinalis* L. At the same time, the alcoholic tinctures of the plants belonging to this family exhibited no nematocidal effects in relation to the invasive nematode larvae (Table 2). The ethanolic tinctures of medicinal plants of the Rosaceae and Fabaceae families had no effect on the vitality of the nematode larvae.

The strongest negative effect on the vitality of the larval stages of the ruminant nematodes in the in vitro conditions was recorded for *Populus nigra* L. (Salicaceae). Over 90% of the L_1–2_ specimens of *S. papillosus* died within a day when subject to the alcohol tincture of this plant (even at 1% concentration of the solution, Table 3). The invasive stages of the nematodes were resistant to the ethanolic tinctures of the plants belonging to the families Salicaceae, Fagaceae, Betulaceae, Juglandaceae, and Rhamnaceae at the same concentrations.

No notable changes in the vitality of the larvae of *S. papillosus* and *H. contortus* in the in vitro conditions were produced by the alcoholic tinctures of the medicinal plants from the families Acoraceae, Apiaceae, Caprifoliaceae, Cucurbitaceae, Equisetaceae, Ericaceae, Gentianaceae, Hypericaceae, Malvaceae, Plantaginaceae, Poaceae, Polygonaceae, and Ranunculaceae (Table 4). Over 50% of the larvae at various stages of development remained vital for 24 h. Only when exposed to *Althaea officinalis* L. (Malvaceae) rhizomes was a slight anthelmintic effect observed: the death of more than 44% of L_1–2_ of *S. papillosus* (non-infection larvae).

## 4. Discussion

All the species we analyzed in this study are traditional medicinal plants, used in folk medicine from ancient times. Most of them exert anti-inflammatory, antiseptic, antimicrobial, and other therapeutic properties that benefit humans and vertebrates [34,35,36]. Due to their bioactive compounds, almost all those plants are used to treat various ailments, including respiratory diseases caused by pathogenic microorganisms and parasitic diseases [37,38].

However, not all of our studies confirmed the results reported by other researchers. According to Tariq et al. [34], aqueous and ethanolic extracts from *Achillea millifolium* exerted notable anthelmintic properties against nematodes of the gastrointestinal tract of ruminants, particularly the mature specimens of *Haemonchus contortus*, in both in vitro and in vivo conditions. According to the in vitro studies, LC_50_ equaled 0.05 and 0.11 mg/mL for aqueous and ethanolic extracts, respectively. The experiments of other researchers testing *A. millifolium* against gastrointestinal nematodes of donkeys confirmed an anthelmintic effect of an aqueous extract of this plant in in vitro conditions, with ovicidal and larvicidal activities [39]. However, our experiment with ethanolic tincture in studied concentrations revealed no negative effect on the nematode larvae of ruminants.

A notable anthelmintic effect was exhibited by the ethanolic tincture of *Artemisia absinthium* on the L_1–2_ larvae of *S. papillosus*. The results of our experiments partially confirmed Tariq et al. [40], who studied the effects of ethanolic extract from *A. absinthium* on the survival of adult nematodes of *H. contortus* in in vitro conditions, and also their egg output in feces. Although this tincture had no effect on the larvae of *H. contortus*, it exhibited a nematocidal effect against the pathogens of strongyloidiasis in ruminants. Our previous studies of anthelmintic properties of ethanolic extracts of plants of the Asteraceae family, including the *Artemisia* genus, revealed no notable effect of a 0.1% concentration, as also evidenced in the experiment conducted by Zazharskyi et al. [41].

In in vitro conditions, the ethanolic tincture of *Calendula officinalis* had no negative effect on the survival of the nematode larvae of ruminants. By contrast, the experiment by Băieş et al. [10], conducted in vivo, revealed a pronounced anthelmintic effect against the eggs of swine nematodes.

According to Buza et al. [42], *Inula helenium* had a significant effect (*p* < 0.01) on the egg output and the development of nematode larvae of donkeys, reporting its anthelmintic activity (LC_50_ 0.041 mg/mL for egg mortality and 0.41 mg/mL for larval mortality). Our findings align with theirs, as the ethanolic extract of *I. helenium* killed over 65% of the first- and second-stage larvae of *S. papillosus* in 24 h.

Thanks to phenols, some species of chamomile have anthelmintic properties [43]. According to our studies, the chamomile *Matricaria chamomilla* produced a negative effect on the nematode larvae of *S. papillosus*. The leaves and flowers of another representative of Asteraceae—*Tanacetum vulgare*—displayed a notable larvicidal action toward the nematode larvae targeting sheep [44]. Their ethanolic extracts at concentrations of 500, 200, 100, 50, 20, and 10 mg/mL exerted larvicidal activity against Trichostrongylidae helminths infecting sheep. The aqueous solutions of the 10% ethanolic tincture at concentrations of 0.1%, 1.0%, and 10% had no effect on different development stages of *H. contortus* and *S. papillosus* in the environment. Compared with the results of our previous studies, the 3% aqueous extracts of *Taraxacum officinale* and *Sanguisorba officinalis* had a stronger negative effect on the survival of nematode larvae of ruminants than the 1% aqueous solutions of ethanolic tinctures [32].

Bioactive compounds of essential oils of *Mentha × piperita*, *Origanum vulgare*, and *Salvia officinalis* have manifested anthelmintic properties against intestinal larvae of ruminants [45,46,47]. However, the alcoholic tinctures of *Mentha × piperita* and *Origanum vulgare* at a 1% concentration had no effect on the nematode larvae of ruminants. Meanwhile, the tincture of *Salvia officinalis* exerted a considerable effect on the larvae of *S. papillosus*. Ferreira et al. [48] confirmed the effect of the essential oil of *Thymus vulgaris* on *H. contortus*. The main component of the essential oil of this plant is thymol. The results of our previous studies [49,50] of plant components on the survival of nematode larvae in in vitro conditions also corroborated that thymol, and its mixtures with other plant components at various concentrations (0.01–1.0%), exhibit negative effects on the larvae of *H. contortus*. However, in the form of 1% aqueous tincture, this plant had no negative effect on the larvae of the studied nematode. In our previous studies of nematocidal activities of alcoholic extracts from medicinal plants, an insignificant effect on the vitality of nematode larvae was produced by plants of the Lamiaceae family, particularly *Stachys recta* at a 0.1% concentration. However, the mortality of the larvae of *S. papillosus* after 24 h exposure to this extract did not exceed 46.9% on average [51].

The root of *Glycyrrhiza glabra* in the form of aqueous extract manifested a negative effect on nematodes of the gastrointestinal tract of sheep [52], as revealed by the egg hatch and development tests on the larvae of *Trichostrongylus* spp. and *Teladorsagia*/*Ostertagia* spp. (LC_50_ measured 12.25 mg/mL). However, the 1% ethanolic tincture of the root demonstrated no effect on any of the studied nematodes.

The aqueous extract of *Quercus robur* in a dose of 3.75 g/kg reduced the fecal egg count of alimentary-canal nematodes from sheep [24]. However, in the in vitro experiment, the 1% ethanolic extract of this plant showed no effect on the nematode larvae.

The in vitro studies revealed a considerable anthelmintic activity of unpurified ethanolic extract of *Juglans regia* on the locomotor activity of adult nematodes of *Ascaridia galli*. At a 100 mg/mL concentration, this extract caused a 96.5% inhibition of the nematodes’ locomotor activity in 24 h [53]. The ethanolic tincture at a 1% concentration, according to our studies, had no notable anthelmintic effect on the nematode larvae.

At a 1% concentration, the ethanolic tinctures of medicinal plants of the Acoraceae, Apiaceae, Caprifoliaceae, Cucurbitaceae, Equisetaceae, Ericaceae, Gentianaceae, Hypericaceae, Malvaceae, Plantaginaceae, Poaceae, Polygonaceae, and Ranunculaceae families demonstrated no notable anthelmintic properties. Nonetheless, there is scientific evidence of their efficacy in other therapeutic forms and concentrations against nematodes parasitizing ruminants. In the in vitro studies, aqueous and ethanolic extracts of the seeds of *Nigella sativa* L. at concentrations of 3.12, 6.3, 12.5, 25.0, and 50.0 mg/mL exhibited an ovicidal effect (*p* < 0.05) on the eggs of gastrointestinal nematodes of ruminants [54].

## 5. Conclusions

The effects of plants on nematodes depend on a number of factors, including the medical form (aqueous tincture, ethanolic extract, etc.), the part of the plant that is used for the preparation of extract or tincture, the base of medical form (water, ethyl acetate, methanol, and other), its concentration, and the target species of parasite and stage of its development (egg, larvae, mature individual). Our studies revealed notable anthelmintic effects of 1% aqueous tinctures of *Artemisia absinthium*, *Inula helenium*, *Matricaria chamomilla*, *Salvia officinalis*, and *Populus nigra* against noninvasive larvae of *S. papillosus*. Over 65% of those larvae died during a 24 h exposure to alcoholic tinctures of these plants. Further in vitro and in vivo studies are needed to determine optimal concentrations of these solutions for future applications for the treatment of animals with gastrointestinal nematodes, including strongyloidiasis.

## Figures and Tables

**Table 1:** Mortality of the larvae of *S. papillosus* and *H. contortus* (%) during a 24 h laboratory experiment under the influence of aqueous solutions of alcoholic tinctures of plants belonging to the Asteraceae family (x ± SD, n = 5).

Plant	Nematode Species	Mortality ^1^ of Nematode Larvae in Control, %	Mortality of Nematode Larvae in 0.01% Solution, %	Mortality of Nematode Larvae in 0.1% Solution, %	Mortality of Nematode Larvae in 1.0% Solution, %
*Achillea millefolium* L.	L_1–2_ of *S. papillosus*	18.1 ± 3.3 ^a^	18.0 ± 4.2 ^a^	20.5 ± 0.9 ^a^	33.7 ± 3.3 ^b^
(Asteraceae)	L_3_ of *S. papillosus*	15.2 ± 3.2 ^a^	14.5 ± 2.7 ^a^	14.3 ± 2.5 ^a^	15.0 ± 4.7 ^a^
Inflorescences	L_3_ of *H. contortus*	0.0 ± 0.0 ^a^	0.0 ± 0.0 ^a^	0.0 ± 0.0 ^a^	0.0 ± 0.0 ^a^
*Arctium lappa* L.	L_1–2_ of *S. papillosus*	11.3 ± 2.4 ^a^	14.2 ± 9.0 ^a^	12.3 ± 3.5 ^a^	16.4 ± 3.0 ^a^
(Asteraceae)	L_3_ of *S. papillosus*	11.2 ± 1.3 ^a^	11.7 ± 5.2 ^a^	11.5 ± 7.1 ^a^	12.4 ± 2.0 ^a^
Rhizomes	L_3_ of *H. contortus*	0.0 ± 0.0 ^a^	2.4 ± 5.3 ^a^	2.0 ± 4.5 ^a^	2.5 ± 5.6 ^a^
*Artemisia absinthium* L.	L_1–2_ of *S. papillosus*	1.5 ± 3.4 ^a^	0.0 ± 0.0 ^a^	1.8 ± 4.1 ^a^	85.5 ± 16.5 ^b^
(Asteraceae)	L_3_ of *S. papillosus*	14.1 ± 4.1 ^a^	15.6 ± 6.7 ^a^	14.5 ± 3.2 ^a^	19.4 ± 6.7 ^a^
Aboveground part	L_3_ of *H. contortus*	0.0 ± 0.0 ^a^	0.0 ± 0.0 ^a^	0.0 ± 0.0 ^a^	0.0 ± 0.0 ^a^
*Bidens tripartita* L.	L_1–2_ of *S. papillosus*	9.4 ± 6.3 ^a^	13.8 ± 13.0 ^a^	13.6 ± 11.6 ^a^	16.7 ± 12.4 ^a^
(Asteraceae)	L_3_ of *S. papillosus*	0.0 ± 0.0 ^a^	0.0 ± 0.0 ^a^	0.0 ± 0.0 ^a^	0.0 ± 0.0 ^a^
Aboveground part	L_3_ of *H. contortus*	0.0 ± 0.0 ^a^	0.0 ± 0.0 ^a^	0.0 ± 0.0 ^a^	0.0 ± 0.0 ^a^
*Calendula officinalis* L.	L_1–2_ of *S. papillosus*	6.3 ± 0.6 ^a^	5.4 ± 2.5 ^a^	6.3 ± 0.9 ^a^	32.2 ± 11.1 ^b^
(Asteraceae)	L_3_ of *S. papillosus*	11.7 ± 4.6 ^a^	10.4 ± 5.5 ^a^	13.9 ± 4.1 ^a^	14.3 ± 2.8 ^a^
Inflorescences	L_3_ of *H. contortus*	0.0 ± 0.0 ^a^	0.0 ± 0.0 ^a^	0.0 ± 0.0 ^a^	8.7 ± 8.1 ^a^
*Cynara cardunculus*	L_1–2_ of *S. papillosus*	17.0 ± 3.3 ^a^	18.1 ± 5.8 ^a^	18.3 ± 7.2 ^a^	17.1 ± 1.4 ^a^
var. *scolymus* L. (Asteraceae)	L_3_ of *S. papillosus*	14.1 ± 2.0 ^a^	14.1 ± 9.4 ^a^	15.7 ± 10.6 ^a^	16.1 ± 3.2 ^a^
Inflorescences	L_3_ of *H. contortus*	0.0 ± 0.0 ^a^	3.3 ± 4.6 ^a^	3.8 ± 5.6 ^a^	2.9 ± 6.4 ^a^
*Echinacea purpurea* (L.)	L_1–2_ of *S. papillosus*	6.3 ± 0.6 ^a^	7.2 ± 3.3 ^a^	31.8 ± 3.5 ^a^	55.3 ± 7.7 ^b^
Moench (Asteraceae)	L_3_ of *S. papillosus*	0.0 ± 0.0 ^a^	0.0 ± 0.0 ^a^	0.0 ± 0.0 ^a^	3.3 ± 7.5 ^a^
Inflorescences	L_3_ of *H. contortus*	0.0 ± 0.0 ^a^	0.0 ± 0.0 ^a^	0.0 ± 0.0 ^a^	23.3 ± 13.7 ^b^
*Echinacea purpurea* (L.)	L_1–2_ of *S. papillosus*	6.3 ± 0.6 ^a^	6.5 ± 1.2 ^a^	5.2 ± 0.6 ^a^	8.3 ± 2.3 ^a^
Moench (Asteraceae)	L_3_ of *S. papillosus*	2.2 ± 5.0 ^a^	0.0 ± 0.0 ^a^	0.0 ± 0.0 ^a^	0.0 ± 0.0 ^a^
Rhizomes	L_3_ of *H. contortus*	3.3 ± 7.5 ^a^	0.0 ± 0.0 ^a^	0.0 ± 0.0 ^a^	2.9 ± 6.4 ^a^
*Helichrysum arenarium* (L.)	L_1–2_ of *S. papillosus*	2.2 ± 5.0 ^a^	4.3 ± 6.0 ^a^	25.5 ± 12.8 ^b^	32.2 ± 13.5 ^b^
Moench (Asteraceae)	L_3_ of *S. papillosus*	0.0 ± 0.0 ^a^	0.0 ± 0.0 ^a^	0.0 ± 0.0 ^a^	0.0 ± 0.0 ^a^
Inflorescences	L_3_ of *H. contortus*	0.0 ± 0.0 ^a^	0.0 ± 0.0 ^a^	0.0 ± 0.0 ^a^	0.0 ± 0.0 ^a^
*Inula helenium* L.	L_1–2_ of *S. papillosus*	20.7 ± 2.0 ^a^	18.5 ± 3.7 ^a^	55.5 ± 10.1 ^b^	67.7 ± 1.0 ^c^
(Asteraceae)	L_3_ of *S. papillosus*	20.8 ± 1.8 ^a^	21.9 ± 5.4 ^a^	22.2 ± 2.9 ^a^	42.8 ± 4.3 ^b^
Rhizomes	L_3_ of *H. contortus*	0.0 ± 0.0 ^a^	6.2 ± 9.1 ^a^	11.5 ± 11.2 ^a^	57.2 ± 19.1 ^b^
*Matricaria chamomilla* L.	L_1–2_ of *S. papillosus*	2.2 ± 5.0 ^a^	33.6 ± 8.0 ^b^	40.4 ± 4.4 ^b^	77.6 ± 12.8 ^c^
(Asteraceae)	L_3_ of *S. papillosus*	2.0 ± 4.5 ^a^	0.0 ± 0.0 ^a^	0.0 ± 0.0 ^a^	0.0 ± 0.0 ^a^
Inflorescences	L_3_ of *H. contortus*	0.0 ± 0.0 ^a^	0.0 ± 0.0 ^a^	0.0 ± 0.0 ^a^	0.0 ± 0.0 ^a^
*Silybum marianum* (L.)	L_1–2_ of *S. papillosus*	17.0 ± 3.3 ^a^	16.7 ± 10.1 ^a^	16.3 ± 7.3 ^a^	16.4 ± 3.6 ^a^
Gaertn. (Asteraceae)	L_3_ of *S. papillosus*	14.1 ± 2.0 ^a^	16.9 ± 7.5 ^a^	16.5 ± 3.3 ^a^	16.0 ± 2.3 ^a^
Seeds	L_3_ of *H. contortus*	0.0 ± 0.0 ^a^	0.0 ± 0.0 ^a^	0.0 ± 0.0 ^a^	0.0 ± 0.0 ^a^
*Tanacetum vulgare* L.	L_1–2_ of *S. papillosus*	15.1 ± 3.9 ^a^	19.2 ± 8.3 ^ab^	25.5 ± 7.1 ^ab^	26.2 ± 2.4 ^b^
(Asteraceae)	L_3_ of *S. papillosus*	0.0 ± 0.0 ^a^	6.6 ± 6.0 ^ab^	8.7 ± 5.9 ^ab^	10.4 ± 6.4 ^b^
Inflorescences	L_3_ of *H. contortus*	0.0 ± 0.0 ^a^	0.0 ± 0.0 ^a^	0.0 ± 0.0 ^a^	0.0 ± 0.0 ^a^
*Taraxacum officinale* (L.) Webb ex F.H.Wigg.	L_1–2_ of *S. papillosus*	7.3 ± 1.0 ^a^	7.2 ± 1.2 ^a^	7.3 ± 1.3 ^a^	16.2 ± 4.4 ^b^
(Asteraceae)	L_3_ of *S. papillosus*	13.3 ± 4.5 ^a^	10.2 ± 7.8 ^a^	16.9 ± 4.1 ^a^	16.4 ± 4.6 ^a^
Roots	L_3_ of *H. contortus*	0.0 ± 0.0 ^a^	0.0 ± 0.0 ^a^	0.0 ± 0.0 ^a^	5.6 ± 7.9 ^a^
*Tragopogon porrifolius* L.	L_1–2_ of *S. papillosus*	21.5 ± 3.4 ^ab^	19.7 ± 6.4 ^a^	22.8 ± 12.5 ^ab^	30.5 ± 8.7 ^b^
(Asteraceae)	L_3_ of *S. papillosus*	17.0 ± 3.2 ^a^	17.7 ± 11.7 ^a^	17.4 ± 16.1 ^a^	15.0 ± 14.6 ^a^
Rhizomes	L_3_ of *H. contortus*	0.0 ± 0.0 ^a^	0.0 ± 0.0 ^a^	0.0 ± 0.0 ^a^	0.0 ± 0.0 ^a^

^1^ The following parts were tested: roots, rhizomes, bark, aboveground part, buds, leaves, inflorescences, pericarp, stamens, fruits, and seeds. Albendazole was used as a positive control: in the positive control, larval mortality after 24 h was 100%. Different letters in Table 1 within each line indicate significant (*p* < 0.05) differences between groups according to Tukey’s test results.

**Table 2:** Mortality of the larvae of *S. papillosus* and *H. contortus* (%) during a 24 h laboratory experiment under the influence of aqueous solutions of alcoholic tinctures of plants belonging to the Rosaceae, Lamiaceae, and Fabaceae families (x ± SD, n = 5).

Plant	Nematode Species	Mortality ^1^ of Nematode Larvae in Control, %	Mortality of Nematode Larvae in 0.01% Solution, %	Mortality of Nematode Larvae in 0.1% Solution, %	Mortality of Nematode Larvae in 1.0% Solution, %
*Agrimonia eupatoria* L.	L_1–2_ of *S. papillosus*	24.5 ± 5.6 ^a^	23.5 ± 12.5 ^a^	23.8 ± 12.7 ^a^	27.2 ± 2.7 ^a^
(Rosaceae)	L_3_ of *S. papillosus*	0.0 ± 0.0 ^a^	7.4 ± 8.0 ^a^	6.7 ± 6.2 ^a^	8.3 ± 8.6 ^a^
Aboveground part	L_3_ of *H. contortus*	2.5 ± 5.6 ^a^	3.1 ± 4.3 ^a^	4.2 ± 5.8 ^a^	4.0 ± 8.9 ^a^
*Fragaria vesca* L.	L_1–2_ of *S. papillosus*	21.9 ± 8.0 ^a^	22.1 ± 7.8 ^a^	24.6 ± 4.0 ^a^	18.1 ± 12.3 ^a^
(Rosaceae)	L_3_ of *S. papillosus*	7.3 ± 10.1 ^a^	8.7 ± 8.3 ^a^	9.5 ± 10.4 ^a^	9.0 ± 12.4 ^a^
Leaves	L_3_ of *H. contortus*	0.0 ± 0.0 ^a^	0.0 ± 0.0 ^a^	0.0 ± 0.0 ^a^	0.0 ± 0.0 ^a^
*Sanguisorba officinalis* L.	L_1–2_ of *S. papillosus*	17.0 ± 3.3 ^a^	17.8 ± 3.9 ^a^	17.6 ± 5.2 ^a^	15.4 ± 3.3 ^a^
(Rosaceae)	L_3_ of *S. papillosus*	14.1 ± 2.0 ^a^	17.8 ± 8.1 ^a^	16.8 ± 14.3 ^a^	17.8 ± 2.3 ^a^
Rhizomes	L_3_ of *H. contortus*	0.0 ± 0.0 ^a^	0.0 ± 0.0 ^a^	0.0 ± 0.0 ^a^	0.0 ± 0.0 ^a^
*Leonurus cardiaca* L.	L_1–2_ of *S. papillosus*	11.3 ± 2.4 ^a^	15.2 ± 8.8 ^a^	14.8 ± 6.0 ^a^	16.1 ± 3.0 ^a^
(Lamiaceae)	L_3_ of *S. papillosus*	11.2 ± 1.3 ^a^	16.4 ± 6.2 ^ab^	16.8 ± 11.0 ^ab^	19.1 ± 2.5 ^b^
Aboveground part	L_3_ of *H. contortus*	0.0 ± 0.0 ^a^	6.3 ± 4.9 ^a^	5.5 ± 5.6 ^a^	6.7 ± 6.2 ^a^
*Mentha × piperita* L.	L_1–2_ of *S. papillosus*	8.3 ± 11.8 ^a^	0.0 ± 0.0 ^a^	7.8 ± 7.5 ^a^	39.4 ± 17.1 ^b^
(Lamiaceae)	L_3_ of *S. papillosus*	4.0 ± 8.9 ^a^	0.0 ± 0.0 ^a^	0.0 ± 0.0 ^a^	0.0 ± 0.0 ^a^
Aboveground part	L_3_ of *H. contortus*	0.0 ± 0.0 ^a^	0.0 ± 0.0 ^a^	0.0 ± 0.0 ^a^	0.0 ± 0.0 ^a^
*Origanum vulgare* L.	L_1–2_ of *S. papillosus*	21.5 ± 3.4 ^a^	19.9 ± 12.2 ^a^	20.4 ± 12.2 ^a^	24.1 ± 6.7 ^a^
(Lamiaceae)	L_3_ of *S. papillosus*	17.0 ± 3.2 ^a^	18.9 ± 6.8 ^a^	17.9 ± 9.8 ^a^	23.5 ± 6.1 ^a^
Aboveground part	L_3_ of *H. contortus*	0.0 ± 0.0 ^a^	5.1 ± 7.0 ^a^	6.4 ± 7.5 ^a^	5.6 ± 7.9 ^a^
*Salvia officinalis* L.	L_1–2_ of *S. papillosus*	1.8 ± 4.1 ^a^	1.5 ± 3.4 ^a^	51.4 ± 10.7 ^b^	80.4 ± 7.0 ^c^
(Lamiaceae)	L_3_ of *S. papillosus*	12.9 ± 5.1 ^ab^	9.6 ± 6.4 ^ab^	10.8 ± 3.2 ^a^	20.7 ± 4.8 ^b^
Aboveground part	L_3_ of *H. contortus*	0.0 ± 0.0 ^a^	0.0 ± 0.0 ^a^	0.0 ± 0.0 ^a^	17.9 ± 6.0 ^a^
*Thymus vulgaris* L.	L_1–2_ of *S. papillosus*	2.2 ± 5.0 ^a^	2.9 ± 6.4 ^a^	6.2 ± 8.5 ^a^	5.4 ± 7.4 ^a^
(Lamiaceae)	L_3_ of *S. papillosus*	15.2 ± 2.6 ^ab^	11.3 ± 1.8 ^a^	12.3 ± 1.4 ^a^	23.9 ± 5.6 ^b^
Aboveground part	L_3_ of *H. contortus*	0.0 ± 0.0 ^a^	0.0 ± 0.0 ^a^	0.0 ± 0.0 ^a^	0.0 ± 0.0 ^a^
*Glycyrrhiza glabra* L.	L_1–2_ of *S. papillosus*	1.4 ± 3.2 ^a^	2.8 ± 4.2 ^a^	4.4 ± 4.0 ^a^	21.9 ± 9.5 ^b^
(Fabaceae)	L_3_ of *S. papillosus*	4.0 ± 8.9 ^a^	8.4 ± 8.5 ^a^	9.8 ± 9.4 ^a^	6.7 ± 6.2 ^a^
Rhizomes	L_3_ of *H. contortus*	0.0 ± 0.0 ^a^	0.0 ± 0.0 ^a^	0.0 ± 0.0 ^a^	6.2 ± 8.5 ^a^
*Hedysarum alpinum* L.	L_1–2_ of *S. papillosus*	15.4 ± 10.2 ^a^	19.7 ± 10.1 ^a^	25.1 ± 11.9 ^a^	25.6 ± 4.7 ^a^
(Fabaceae)	L_3_ of *S. papillosus*	4.7 ± 6.5 ^a^	6.8 ± 6.8 ^a^	6.7 ± 6.2 ^a^	6.9 ± 9.6 ^a^
Rhizomes	L_3_ of *H. contortus*	0.0 ± 0.0 ^a^	0.0 ± 0.0 ^a^	0.0 ± 0.0 ^a^	0.0 ± 0.0 ^a^
*Trifolium pratense* L.	L_1–2_ of *S. papillosus*	15.7 ± 4.3 ^a^	20.5 ± 7.5 ^ab^	28.7 ± 7.7 ^b^	35.0 ± 4.1 ^b^
(Fabaceae)	L_3_ of *S. papillosus*	0.0 ± 0.0 ^a^	0.0 ± 0.0 ^a^	0.0 ± 0.0 ^a^	0.0 ± 0.0 ^a^
Inflorescences	L_3_ of *H. contortus*	0.0 ± 0.0 ^a^	1.4 ± 3.2 ^a^	1.3 ± 2.8 ^a^	1.3 ± 2.8 ^a^

^1^ See Table 1.

**Table 3:** Mortality of the larvae of *S. papillosus* and *H. contortus* (%) during a 24 h laboratory experiment under the influence of aqueous solutions of alcoholic tinctures of tree plants belonging to the families Salicaceae, Fagaceae, Betulaceae, Juglandaceae, and Rhamnaceae (x ± SD, n = 5).

Plant	Nematode Species	Mortality ^1^ of Nematode Larvae in Control, %	Mortality of Nematode Larvae in 0.01% Solution, %	Mortality of Nematode Larvae in 0.1% Solution, %	Mortality of Nematode Larvae in 1.0% Solution, %
*Populus nigra* L.	L_1–2_ of *S. papillosus*	2.4 ± 2.0 ^a^	25.2 ± 7.0 ^b^	90.0 ± 1.9 ^c^	93.2 ± 1.6 ^c^
(Salicaceae)	L_3_ of *S. papillosus*	13.5 ± 3.0 ^a^	12.4 ± 3.9 ^a^	11.6 ± 3.6 ^a^	25.5 ± 5.3 ^b^
Buds	L_3_ of *H. contortus*	0.0 ± 0.0 ^a^	0.0 ± 0.0 ^a^	0.0 ± 0.0 ^a^	2.2 ± 5.0 ^a^
*Populus tremula* L.	L_1–2_ of *S. papillosus*	11.6 ± 3.4 ^a^	15.0 ± 5.1 ^ab^	16.6 ± 5.5 ^ab^	24.9 ± 5.1 ^b^
(Salicaceae)	L_3_ of *S. papillosus*	9.3 ± 3.1 ^a^	9.7 ± 2.4 ^a^	9.3 ± 3.6 ^a^	20.4 ± 4.4 ^b^
Bark	L_3_ of *H. contortus*	0.0 ± 0.0 ^a^	0.0 ± 0.0 ^a^	0.0 ± 0.0 ^a^	4.8 ± 4.6 ^a^
*Salix alba* L.	L_1–2_ of *S. papillosus*	7.3 ± 1.0 ^a^	7.4 ± 2.1 ^a^	5.1 ± 1.0 ^a^	7.1 ± 1.3 ^a^
(Salicaceae)	L_3_ of *S. papillosus*	13.6 ± 2.8 ^a^	13.1 ± 2.6 ^a^	12.7 ± 4.4 ^a^	28.3 ± 3.7 ^b^
Bark	L_3_ of *H. contortus*	0.0 ± 0.0 ^a^	0.0 ± 0.0 ^a^	0.0 ± 0.0 ^a^	0.0 ± 0.0 ^a^
*Quercus robur* L.	L_1–2_ of *S. papillosus*	6.3 ± 0.6 ^ab^	6.2 ± 0.2 ^ab^	5.5 ± 1.3 ^a^	10.9 ± 3.8 ^b^
(Fagaceae)	L_3_ of *S. papillosus*	0.0 ± 0.0 ^a^	0.0 ± 0.0 ^a^	0.0 ± 0.0 ^a^	9.2 ± 5.5 ^b^
Bark	L_3_ of *H. contortus*	2.5 ± 5.6 ^a^	2.2 ± 5.0 ^a^	2.0 ± 4.5 ^a^	2.9 ± 6.4 ^a^
*Betula pendula* Roth	L_1–2_ of *S. papillosus*	17.0 ± 3.3 ^a^	17.2 ± 5.2 ^a^	17.9 ± 9.0 ^a^	17.5 ± 1.9 ^a^
(Betulaceae)	L_3_ of *S. papillosus*	14.1 ± 2.0 ^a^	14.9 ± 8.9 ^a^	15.7 ± 4.0 ^a^	14.9 ± 2.0 ^a^
Leaves	L_3_ of *H. contortus*	0.0 ± 0.0 ^a^	0.0 ± 0.0 ^a^	0.0 ± 0.0 ^a^	0.0 ± 0.0 ^a^
*Betula pendula* Roth	L_1–2_ of *S. papillosus*	10.2 ± 6.1 ^a^	8.8 ± 8.4 ^a^	10.5 ± 6.4 ^a^	11.0 ± 10.1 ^a^
(Betulaceae)	L_3_ of *S. papillosus*	0.0 ± 0.0 ^a^	0.0 ± 0.0 ^a^	0.0 ± 0.0 ^a^	0.0 ± 0.0 ^a^
Buds	L_3_ of *H. contortus*	0.0 ± 0.0 ^a^	0.0 ± 0.0 ^a^	0.0 ± 0.0 ^a^	0.0 ± 0.0 ^a^
*Juglans regia* L.	L_1–2_ of *S. papillosus*	21.9 ± 8.0 ^a^	19.5 ± 6.3 ^a^	19.9 ± 5.7 ^a^	19.1 ± 5.7 ^a^
(Juglandaceae)	L_3_ of *S. papillosus*	5.0 ± 7.5 ^a^	6.7 ± 6.2 ^a^	7.4 ± 9.1 ^a^	9.8 ± 9.9 ^a^
Leaves	L_3_ of *H. contortus*	0.0 ± 0.0 ^a^	0.0 ± 0.0 ^a^	0.0 ± 0.0 ^a^	0.0 ± 0.0 ^a^
*Frangula alnus* Mill.	L_1–2_ of *S. papillosus*	21.5 ± 3.4 ^a^	22.9 ± 12.1 ^a^	19.4 ± 4.8 ^a^	20.3 ± 2.2 ^a^
(Rhamnaceae)	L_3_ of *S. papillosus*	17.0 ± 3.2 ^a^	16.7 ± 8.9 ^a^	19.3 ± 12.3 ^a^	17.3 ± 5.4 ^a^
Fruits	L_3_ of *H. contortus*	0.0 ± 0.0 ^a^	2.6 ± 3.6 ^a^	3.5 ± 4.9 ^a^	4.3 ± 6.0 ^a^

^1^ See Table 1.

**Table 4:** Mortality of the larvae of *S. papillosus* and *H. contortus* (%) during 24 h laboratory experiment under the influence of aqueous solutions of alcoholic tinctures of tree plants belonging to the families Acoraceae, Apiaceae, Caprifoliaceae, Cucurbitaceae, Equisetaceae, Ericaceae, Gentianaceae, Hypericaceae, Malvaceae, Plantaginaceae, Poaceae, Polygonaceae, and Ranunculaceae (x ± SD, n = 5).

Plant	Nematode Species	Mortality ^1^ of Nematode Larvae in Control, %	Mortality of Nematode Larvae in 0.01% Solution, %	Mortality of Nematode Larvae in 0.1% Solution, %	Mortality of Nematode Larvae in 1.0% Solution, %
*Acorus calamus* L.	L_1–2_ of *S. papillosus*	24.5 ± 5.6 ^a^	29.5 ± 6.7 ^a^	26.8 ± 8.1 ^a^	31.2 ± 6.8 ^a^
(Acoraceae)	L_3_ of *S. papillosus*	0.0 ± 0.0 ^a^	3.7 ± 5.2 ^a^	1.4 ± 3.2 ^a^	1.7 ± 3.7 ^a^
Rhizomes	L_3_ of *H. contortus*	2.5 ± 5.6 ^a^	5.2 ± 7.1 ^a^	9.0 ± 8.5 ^a^	6.1 ± 5.8 ^a^
*Foeniculum vulgare* Mill.	L_1–2_ of *S. papillosus*	21.9 ± 8.0 ^a^	20.1 ± 5.9 ^a^	22.0 ± 10.5 ^a^	19.8 ± 3.4 ^a^
(Apiaceae)	L_3_ of *S. papillosus*	5.3 ± 7.7 ^a^	11.7 ± 9.3 ^a^	10.2 ± 9.9 ^a^	11.7 ± 7.2 ^a^
Fruits	L_3_ of *H. contortus*	0.0 ± 0.0 ^a^	0.0 ± 0.0 ^a^	0.0 ± 0.0 ^a^	0.0 ± 0.0 ^a^
*Valeriana officinalis* L.	L_1–2_ of *S. papillosus*	6.3 ± 0.6 ^a^	8.1 ± 0.9 ^a^	8.9 ± 2.2 ^a^	32.4 ± 4.1 ^b^
(Caprifoliaceae)	L_3_ of *S. papillosus*	2.9 ± 6.4 ^a^	0.0 ± 0.0 ^a^	0.0 ± 0.0 ^a^	10.6 ± 10.1 ^a^
Rhizomes	L_3_ of *H. contortus*	10.7 ± 15.3 ^a^	2.9 ± 6.4 ^a^	2.5 ± 5.6 ^a^	12.6 ± 12.6 ^a^
*Cucurbita pepo* L.	L_1–2_ of *S. papillosus*	1.4 ± 3.2 ^a^	0.8 ± 1.9 ^a^	1.6 ± 2.2 ^a^	11.5 ± 3.4 ^b^
(Cucurbitaceae)	L_3_ of *S. papillosus*	9.3 ± 3.1 ^ab^	6.5 ± 5.2 ^a^	6.1 ± 5.6 ^a^	15.0 ± 3.4 ^b^
Seeds	L_3_ of *H. contortus*	0.0 ± 0.0 ^a^	0.0 ± 0.0 ^a^	0.0 ± 0.0 ^a^	0.0 ± 0.0 ^a^
*Cucurbita pepo* L.	L_1–2_ of *S. papillosus*	6.3 ± 0.6 ^a^	6.2 ± 1.3 ^a^	6.6 ± 1.4 ^a^	7.0 ± 1.3 ^a^
(Cucurbitaceae)	L_3_ of *S. papillosus*	9.3 ± 3.1 ^a^	9.8 ± 2.9 ^a^	11.9 ± 3.3 ^ab^	16.1 ± 2.5 ^b^
Pericarp	L_3_ of *H. contortus*	0.0 ± 0.0 ^a^	0.0 ± 0.0 ^a^	0.0 ± 0.0 ^a^	6.4 ± 5.9 ^a^
*Equisetum arvense* L.	L_1–2_ of *S. papillosus*	21.5 ± 3.4 ^a^	20.8 ± 10.2 ^a^	23.0 ± 7.1 ^a^	22.2 ± 3.4 ^a^
(Equisetaceae)	L_3_ of *S. papillosus*	17.0 ± 3.2 ^a^	15.1 ± 9.8 ^a^	16.3 ± 11.3 ^a^	18.0 ± 6.8 ^a^
Aboveground part	L_3_ of *H. contortus*	0.0 ± 0.0 ^a^	0.0 ± 0.0 ^a^	0.0 ± 0.0 ^a^	0.0 ± 0.0 ^a^
*Vaccinium vitis-idaea* L.	L_1–2_ of *S. papillosus*	10.2 ± 6.1 ^a^	12.2 ± 8.9 ^a^	12.7 ± 7.3 ^a^	11.2 ± 6.9 ^a^
(Ericaceae)	L_3_ of *S. papillosus*	0.0 ± 0.0 ^a^	0.0 ± 0.0 ^a^	0.0 ± 0.0 ^a^	0.0 ± 0.0 ^a^
Aboveground part	L_3_ of *H. contortus*	0.0 ± 0.0 ^a^	0.0 ± 0.0 ^a^	0.0 ± 0.0 ^a^	0.0 ± 0.0 ^a^
*Centaurium erythraea* Rafn	L_1–2_ of *S. papillosus*	1.4 ± 3.2 ^a^	3.5 ± 5.2 ^a^	4.1 ± 3.9 ^a^	22.2 ± 1.7 ^b^
(Gentianaceae)	L_3_ of *S. papillosus*	9.9 ± 4.4 ^a^	9.6 ± 4.5 ^a^	9.5 ± 3.1 ^a^	30.6 ± 2.5 ^b^
Aboveground part	L_3_ of *H. contortus*	0.0 ± 0.0 ^a^	0.0 ± 0.0 ^a^	0.0 ± 0.0 ^a^	0.0 ± 0.0 ^a^
*Hypericum perforatum* L.	L_1–2_ of *S. papillosus*	15.2 ± 9.4 ^a^	16.7 ± 6.4 ^a^	14.4 ± 9.4 ^a^	17.5 ± 16.8 ^a^
(Hypericaceae)	L_3_ of *S. papillosus*	0.0 ± 0.0 ^a^	0.0 ± 0.0 ^a^	0.0 ± 0.0 ^a^	0.0 ± 0.0 ^a^
Aboveground part	L_3_ of *H. contortus*	0.0 ± 0.0 ^a^	0.0 ± 0.0 ^a^	0.0 ± 0.0 ^a^	0.0 ± 0.0 ^a^
*Althaea officinalis* L.	L_1–2_ of *S. papillosus*	2.2 ± 5.0 ^a^	13.5 ± 8.0 ^ab^	24.2 ± 16.0 ^b^	44.3 ± 26.4 ^b^
(Malvaceae)	L_3_ of *S. papillosus*	21.2 ± 4.3 ^a^	18.7 ± 5.8 ^a^	20.4 ± 3.9 ^a^	22.9 ± 1.9 ^a^
Rhizomes	L_3_ of *H. contortus*	0.0 ± 0.0 ^a^	0.0 ± 0.0 ^a^	0.0 ± 0.0 ^a^	0.0 ± 0.0 ^a^
*Linaria vulgaris* Mill.	L_1–2_ of *S. papillosus*	20.7 ± 2.0 ^a^	19.6 ± 4.9 ^a^	20.0 ± 5.0 ^a^	19.6 ± 2.4 ^a^
(Plantaginaceae)	L_3_ of *S. papillosus*	20.8 ± 1.8 ^a^	20.5 ± 4.2 ^a^	19.8 ± 6.0 ^a^	22.7 ± 4.3 ^a^
Aboveground part	L_3_ of *H. contortus*	0.0 ± 0.0 ^a^	0.0 ± 0.0 ^a^	0.0 ± 0.0 ^a^	0.0 ± 0.0 ^a^
*Plantago major* L.	L_1–2_ of *S. papillosus*	20.7 ± 2.0 ^a^	22.2 ± 3.5 ^a^	19.5 ± 3.5 ^a^	21.9 ± 4.2 ^a^
(Plantaginaceae)	L_3_ of *S. papillosus*	20.8 ± 1.8 ^a^	20.4 ± 7.0 ^a^	21.5 ± 6.9 ^a^	20.9 ± 6.7 ^a^
Leaves	L_3_ of *H. contortus*	0.0 ± 0.0 ^a^	0.0 ± 0.0 ^a^	0.0 ± 0.0 ^a^	0.0 ± 0.0 ^a^
*Zea mays* L.	L_1–2_ of *S. papillosus*	13.7 ± 4.7 ^a^	13.7 ± 3.7 ^a^	11.1 ± 3.5 ^a^	11.4 ± 7.6 ^a^
(Poaceae)	L_3_ of *S. papillosus*	0.0 ± 0.0 ^a^	0.0 ± 0.0 ^a^	0.0 ± 0.0 ^a^	5.4 ± 7.4 ^a^
Stamens	L_3_ of *H. contortus*	0.0 ± 0.0 ^a^	0.0 ± 0.0 ^a^	0.0 ± 0.0 ^a^	0.0 ± 0.0 ^a^
*Polygonum aviculare* L.	L_1–2_ of *S. papillosus*	13.7 ± 4.7 ^a^	14.9 ± 2.2 ^a^	11.7 ± 2.2 ^a^	15.5 ± 3.0 ^a^
(Polygonaceae)	L_3_ of *S. papillosus*	21.0 ± 4.7 ^a^	17.5 ± 4.9 ^a^	20.3 ± 5.6 ^a^	24.1 ± 3.3 ^a^
Aboveground part	L_3_ of *H. contortus*	0.0 ± 0.0 ^a^	0.0 ± 0.0 ^a^	0.0 ± 0.0 ^a^	0.0 ± 0.0 ^a^
*Nigella sativa* L.	L_1–2_ of *S. papillosus*	2.2 ± 5.0 ^a^	0.0 ± 0.0 ^a^	2.5 ± 5.6 ^a^	6.9 ± 9.6 ^a^
(Ranunculaceae)	L_3_ of *S. papillosus*	17.4 ± 2.3 ^a^	17.8 ± 5.2 ^a^	17.5 ± 5.1 ^a^	19.7 ± 2.9 ^a^
Seeds	L_3_ of *H. contortus*	0.0 ± 0.0 ^a^	0.0 ± 0.0 ^a^	0.0 ± 0.0 ^a^	2.5 ± 3.4 ^a^

^1^ See Table 1.

## Data Availability

The data presented in this study are available on request from the corresponding author.
